# *N*′-(3-Bromo-4-methoxy­benzyl­idene)nicotinohydrazide monohydrate

**DOI:** 10.1107/S160053680903400X

**Published:** 2009-08-29

**Authors:** Feng-Yu Bao, Xing-Shun Ding, Hai-Yan Zhang, Ying-Xia Zhou

**Affiliations:** a Department of Applied Chemistry, College of Sciences, Henan Agricultural University, Zhengzhou 450002, People’s Republic of China; b Liangbaosi First School, Jiaxiang County, Shandong Province 272404, People’s Republic of China

## Abstract

In the title compound, C_14_H_12_BrN_3_O_2_·H_2_O, the benzene ring is oriented at a dihedral angle of 39.66 (11)° with respect to the pyridine ring. The solvent water mol­ecule links with the organic compound *via* O—H⋯O, O—H⋯N and N—H⋯O hydrogen bonding.

## Related literature

For applications of Schiff base compounds, see: Kahwa *et al.* (1986 ▶); Santos *et al.* (2001 ▶).
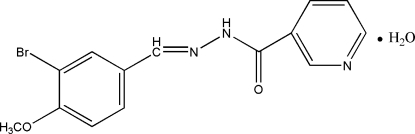


## Experimental

### 

#### Crystal data

C_14_H_12_BrN_3_O_2_·H_2_O
*M* *_r_* = 352.18
Monoclinic, 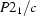

*a* = 7.7979 (1) Å
*b* = 12.5678 (2) Å
*c* = 14.9419 (3) Å
β = 97.449 (1)°
*V* = 1451.98 (4) Å^3^
*Z* = 4
Mo *K*α radiation
μ = 2.85 mm^−1^
*T* = 296 K
0.43 × 0.28 × 0.22 mm

#### Data collection

Bruker SMART CCD area-detector diffractometer
Absorption correction: multi-scan (*SADABS*; Bruker, 1998 ▶) *T*_min_ = 0.400, *T*_max_ = 0.535
12611 measured reflections
3149 independent reflections
2525 reflections with *I* > 2σ(*I*)
*R*_int_ = 0.027

#### Refinement

*R*[*F* ^2^ > 2σ(*F* ^2^)] = 0.027
*wR*(*F* ^2^) = 0.069
*S* = 1.01
3149 reflections
198 parameters
2 restraints
H atoms treated by a mixture of independent and constrained refinement
Δρ_max_ = 0.41 e Å^−3^
Δρ_min_ = −0.30 e Å^−3^

### 

Data collection: *SMART* (Bruker, 1998 ▶); cell refinement: *SAINT* (Bruker, 1998 ▶); data reduction: *SAINT*; program(s) used to solve structure: *SHELXTL* (Sheldrick, 2008 ▶); program(s) used to refine structure: *SHELXTL*; molecular graphics: *SHELXTL*; software used to prepare material for publication: *SHELXTL*.

## Supplementary Material

Crystal structure: contains datablocks global, I. DOI: 10.1107/S160053680903400X/xu2597sup1.cif

Structure factors: contains datablocks I. DOI: 10.1107/S160053680903400X/xu2597Isup2.hkl

Additional supplementary materials:  crystallographic information; 3D view; checkCIF report

## Figures and Tables

**Table 1:** Hydrogen-bond geometry (Å, °)

*D*—H⋯*A*	*D*—H	H⋯*A*	*D*⋯*A*	*D*—H⋯*A*
O2—H2*A*⋯N3	0.85 (2)	2.05 (2)	2.886 (2)	168 (2)
O2—H2*B*⋯O1^i^	0.82 (3)	2.47 (3)	3.118 (2)	136 (2)
O2—H2*B*⋯N1^i^	0.82 (3)	2.43 (3)	3.197 (2)	156 (3)
N2—H2⋯O2^ii^	0.86	2.18	2.982 (2)	155

Symmetry codes: (i) 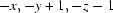; (ii) 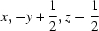.

**Fig. 1.**
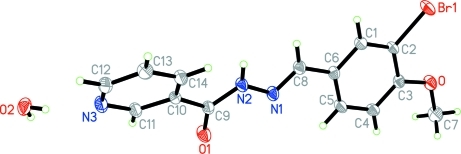
The molecular structure of the title compound. Displacement ellipsoids are drawn at the 30% probability level.

## supplementary crystallographic information

### Comment

The chemistry of Schiff bases has attracted a great deal of interest in recent years. These compounds play an important role in the development of various proteins and enzymes (Kahwa *et al.*, 1986; Santos *et al.*, 2001). As part of our interest in the coordination chemistry of Schiff bases, we have recently synthesized the title compound and report here its crystal structure.

The title molecule crystallizes in the E conformation, with the C8—N1—N2—C9 torsion angle of 170.02 (19)°. The molecular is non-planar, there is a dihedral angle of 39.66 (11)° between the benzene ring and the pyridine ring. In the crystal structure, the lattice water molecule links with the organic compound *via* O—H···O, O—H···N and N—H···O hydrogen bonding.

### Experimental

Nicotinohydrazide (1 mmol, 0.137 g) was dissolved in ethanol (15 ml). The mixture was stirred for several min at 351 K, then 3-bromo-4-methoxybenzaldehyde (1 mmol, 0.215 g) in ethanol (8 mm l) was added dropwise and the mixture was refluxed for 2 h. The solid product was isolated and recrystallized from methanol, colourless single crystals were obtained after 3 d.

### Refinement

H atoms of water molecule are located in a difference Fourier map and refined isotropically, with O—H and H···H distances restrained to 0.85 (1) and 1.37 (2) Å, respectively. Methyl H atoms were placed in calculated positions with C—H = 0.96 Å and refined with *U*_iso_(H) = 1.5*U*_eq_(C). Other H atoms were placed in calculated positions with C—H = 0.93 and N—H = 0.86 Å, and refined in riding mode with *U*_iso_(H) = 1.2*U*_eq_(C,*N*).

### Crystal data

**Table:**

C_14_H_12_BrN_3_O_2_·H_2_O	*F*(000) = 712
*M**_r_* = 352.18	*D*_x_ = 1.611 Mg m^−^^3^
Monoclinic, *P*2_1_/*c*	Mo *K*α radiation, λ = 0.71073 Å
Hall symbol: -P 2ybc	Cell parameters from 4706 reflections
*a* = 7.7979 (1) Å	θ = 3.1–27.0°
*b* = 12.5678 (2) Å	µ = 2.85 mm^−^^1^
*c* = 14.9419 (3) Å	*T* = 296 K
β = 97.449 (1)°	Block, colourless
*V* = 1451.98 (4) Å^3^	0.43 × 0.28 × 0.22 mm
*Z* = 4	

### Data collection

**Table:**

Bruker SMART CCD area-detector diffractometer	3149 independent reflections
Radiation source: fine-focus sealed tube	2525 reflections with *I* > 2σ(*I*)
graphite	*R*_int_ = 0.027
ω scans	θ_max_ = 27.0°, θ_min_ = 3.1°
Absorption correction: multi-scan (*SADABS*; Bruker, 1998)	*h* = −9→9
*T*_min_ = 0.400, *T*_max_ = 0.535	*k* = −16→16
12611 measured reflections	*l* = −19→16

### Refinement

**Table:**

Refinement on *F*^2^	Primary atom site location: structure-invariant direct methods
Least-squares matrix: full	Secondary atom site location: difference Fourier map
*R*[*F*^2^ > 2σ(*F*^2^)] = 0.027	Hydrogen site location: inferred from neighbouring sites
*wR*(*F*^2^) = 0.069	H atoms treated by a mixture of independent and constrained refinement
*S* = 1.01	*w* = 1/[σ^2^(*F*_o_^2^) + (0.0354*P*)^2^ + 0.4288*P*] where *P* = (*F*_o_^2^ + 2*F*_c_^2^)/3
3149 reflections	(Δ/σ)_max_ < 0.001
198 parameters	Δρ_max_ = 0.41 e Å^−^^3^
2 restraints	Δρ_min_ = −0.29 e Å^−^^3^

### Special details

**Table:**

Geometry. All e.s.d.'s (except the e.s.d. in the dihedral angle between two l.s. planes) are estimated using the full covariance matrix. The cell e.s.d.'s are taken into account individually in the estimation of e.s.d.'s in distances, angles and torsion angles; correlations between e.s.d.'s in cell parameters are only used when they are defined by crystal symmetry. An approximate (isotropic) treatment of cell e.s.d.'s is used for estimating e.s.d.'s involving l.s. planes.
Refinement. Refinement of *F*^2^ against ALL reflections. The weighted *R*-factor *wR* and goodness of fit *S* are based on *F*^2^, conventional *R*-factors *R* are based on *F*, with *F* set to zero for negative *F*^2^. The threshold expression of *F*^2^ > σ(*F*^2^) is used only for calculating *R*-factors(gt) *etc*. and is not relevant to the choice of reflections for refinement. *R*-factors based on *F*^2^ are statistically about twice as large as those based on *F*, and *R*- factors based on ALL data will be even larger.

### Fractional atomic coordinates and isotropic or equivalent isotropic displacement parameters (Å^2^)

**Table:**

	*x*	*y*	*z*	*U*_iso_*/*U*_eq_	
Br1	0.38290 (3)	0.882695 (16)	0.139661 (15)	0.04825 (10)	
O1	−0.0836 (2)	0.53946 (11)	−0.37189 (9)	0.0481 (4)	
N1	0.0118 (2)	0.66340 (13)	−0.22564 (10)	0.0364 (4)	
C2	0.2856 (2)	0.77093 (14)	0.06618 (12)	0.0319 (4)	
C6	0.1504 (3)	0.70831 (15)	−0.07779 (12)	0.0330 (4)	
N2	−0.0635 (2)	0.70309 (13)	−0.30777 (10)	0.0373 (4)	
H2	−0.0792	0.7704	−0.3150	0.045*	
C5	0.1501 (3)	0.60611 (15)	−0.04249 (14)	0.0389 (5)	
H5A	0.1064	0.5501	−0.0792	0.047*	
O	0.3473 (2)	0.65767 (12)	0.19064 (9)	0.0467 (4)	
N3	−0.2839 (3)	0.66239 (14)	−0.61976 (11)	0.0445 (4)	
C13	−0.3787 (3)	0.81441 (17)	−0.54493 (14)	0.0416 (5)	
H13A	−0.4406	0.8779	−0.5484	0.050*	
C1	0.2194 (3)	0.79090 (15)	−0.02219 (12)	0.0344 (4)	
H1A	0.2207	0.8598	−0.0448	0.041*	
C14	−0.2933 (3)	0.78026 (16)	−0.46379 (13)	0.0373 (4)	
H14A	−0.2956	0.8207	−0.4118	0.045*	
C12	−0.3718 (3)	0.75404 (18)	−0.62074 (13)	0.0438 (5)	
H12A	−0.4306	0.7777	−0.6752	0.053*	
C11	−0.2044 (3)	0.62932 (16)	−0.54085 (13)	0.0386 (5)	
H11A	−0.1453	0.5649	−0.5392	0.046*	
C10	−0.2041 (2)	0.68508 (15)	−0.46049 (12)	0.0313 (4)	
C3	0.2824 (3)	0.66925 (15)	0.10238 (12)	0.0336 (4)	
C4	0.2141 (3)	0.58670 (16)	0.04669 (13)	0.0386 (5)	
H4A	0.2116	0.5180	0.0696	0.046*	
C8	0.0758 (3)	0.73423 (16)	−0.17017 (13)	0.0373 (4)	
H8A	0.0751	0.8048	−0.1890	0.045*	
C7	0.3529 (4)	0.55397 (18)	0.22835 (15)	0.0573 (7)	
H7A	0.4014	0.5573	0.2907	0.086*	
H7B	0.4233	0.5089	0.1962	0.086*	
H7C	0.2379	0.5255	0.2237	0.086*	
C9	−0.1121 (3)	0.63517 (15)	−0.37660 (12)	0.0332 (4)	
O2	−0.1669 (3)	0.57231 (13)	−0.77890 (10)	0.0521 (4)	
H2A	−0.214 (3)	0.5932 (19)	−0.7336 (13)	0.068 (9)*	
H2B	−0.110 (3)	0.519 (2)	−0.7634 (19)	0.072 (9)*	

### Atomic displacement parameters (Å^2^)

**Table:**

	*U*^11^	*U*^22^	*U*^33^	*U*^12^	*U*^13^	*U*^23^
Br1	0.06384 (18)	0.03325 (12)	0.04243 (13)	−0.00024 (10)	−0.01295 (10)	−0.01048 (9)
O1	0.0686 (11)	0.0340 (8)	0.0379 (8)	0.0075 (7)	−0.0074 (7)	−0.0026 (6)
N1	0.0453 (10)	0.0359 (8)	0.0259 (8)	0.0031 (8)	−0.0035 (7)	0.0004 (7)
C2	0.0353 (11)	0.0284 (9)	0.0298 (9)	−0.0003 (8)	−0.0036 (8)	−0.0066 (7)
C6	0.0352 (11)	0.0358 (10)	0.0265 (9)	0.0022 (8)	−0.0014 (8)	−0.0018 (8)
N2	0.0513 (11)	0.0315 (8)	0.0260 (8)	0.0006 (7)	−0.0065 (7)	0.0007 (6)
C5	0.0460 (13)	0.0336 (10)	0.0345 (10)	−0.0031 (9)	−0.0050 (9)	−0.0054 (8)
O	0.0678 (11)	0.0386 (8)	0.0292 (7)	0.0033 (7)	−0.0105 (7)	0.0033 (6)
N3	0.0600 (12)	0.0418 (10)	0.0293 (8)	−0.0012 (9)	−0.0031 (8)	−0.0037 (7)
C13	0.0437 (13)	0.0374 (10)	0.0419 (11)	0.0047 (9)	−0.0008 (9)	0.0031 (9)
C1	0.0391 (12)	0.0299 (9)	0.0323 (9)	0.0027 (8)	−0.0020 (8)	0.0009 (8)
C14	0.0414 (12)	0.0377 (10)	0.0322 (10)	−0.0007 (9)	0.0021 (8)	−0.0052 (8)
C12	0.0507 (14)	0.0457 (12)	0.0318 (10)	−0.0048 (10)	−0.0074 (9)	0.0049 (9)
C11	0.0499 (13)	0.0342 (10)	0.0300 (10)	−0.0010 (9)	−0.0011 (9)	−0.0027 (8)
C10	0.0328 (11)	0.0329 (9)	0.0270 (9)	−0.0053 (8)	−0.0003 (8)	−0.0013 (7)
C3	0.0371 (11)	0.0350 (10)	0.0270 (9)	0.0045 (9)	−0.0022 (8)	0.0004 (8)
C4	0.0500 (13)	0.0282 (9)	0.0353 (10)	−0.0017 (9)	−0.0028 (9)	0.0032 (8)
C8	0.0429 (12)	0.0372 (10)	0.0299 (9)	0.0001 (9)	−0.0020 (8)	0.0005 (8)
C7	0.0838 (19)	0.0451 (13)	0.0387 (12)	0.0038 (12)	−0.0086 (11)	0.0144 (10)
C9	0.0384 (11)	0.0335 (10)	0.0270 (9)	−0.0006 (8)	0.0016 (8)	−0.0018 (7)
O2	0.0845 (13)	0.0375 (8)	0.0337 (8)	0.0076 (9)	0.0059 (8)	0.0042 (7)

### Geometric parameters (Å, °)

**Table:**

Br1—C2	1.8810 (17)	C13—C14	1.374 (3)
O1—C9	1.224 (2)	C13—H13A	0.9300
N1—C8	1.273 (2)	C1—H1A	0.9300
N1—N2	1.383 (2)	C14—C10	1.382 (3)
C2—C1	1.377 (2)	C14—H14A	0.9300
C2—C3	1.389 (3)	C12—H12A	0.9300
C6—C5	1.389 (3)	C11—C10	1.390 (3)
C6—C1	1.393 (3)	C11—H11A	0.9300
C6—C8	1.463 (3)	C10—C9	1.499 (3)
N2—C9	1.352 (2)	C3—C4	1.392 (3)
N2—H2	0.8600	C4—H4A	0.9300
C5—C4	1.383 (3)	C8—H8A	0.9300
C5—H5A	0.9300	C7—H7A	0.9600
O—C3	1.358 (2)	C7—H7B	0.9600
O—C7	1.418 (3)	C7—H7C	0.9600
N3—C11	1.326 (3)	O2—H2A	0.85 (2)
N3—C12	1.340 (3)	O2—H2B	0.82 (3)
C13—C12	1.370 (3)		
			
C8—N1—N2	114.26 (16)	C13—C12—H12A	118.6
C1—C2—C3	121.17 (17)	N3—C11—C10	123.96 (19)
C1—C2—Br1	119.76 (14)	N3—C11—H11A	118.0
C3—C2—Br1	119.07 (13)	C10—C11—H11A	118.0
C5—C6—C1	118.83 (17)	C14—C10—C11	117.46 (18)
C5—C6—C8	122.96 (17)	C14—C10—C9	125.20 (17)
C1—C6—C8	118.18 (17)	C11—C10—C9	117.30 (17)
C9—N2—N1	119.48 (16)	O—C3—C2	116.98 (17)
C9—N2—H2	120.3	O—C3—C4	124.49 (17)
N1—N2—H2	120.3	C2—C3—C4	118.54 (17)
C4—C5—C6	120.74 (18)	C5—C4—C3	120.46 (18)
C4—C5—H5A	119.6	C5—C4—H4A	119.8
C6—C5—H5A	119.6	C3—C4—H4A	119.8
C3—O—C7	118.23 (16)	N1—C8—C6	122.20 (18)
C11—N3—C12	117.32 (17)	N1—C8—H8A	118.9
C12—C13—C14	119.3 (2)	C6—C8—H8A	118.9
C12—C13—H13A	120.3	O—C7—H7A	109.5
C14—C13—H13A	120.3	O—C7—H7B	109.5
C2—C1—C6	120.25 (17)	H7A—C7—H7B	109.5
C2—C1—H1A	119.9	O—C7—H7C	109.5
C6—C1—H1A	119.9	H7A—C7—H7C	109.5
C13—C14—C10	119.09 (19)	H7B—C7—H7C	109.5
C13—C14—H14A	120.5	O1—C9—N2	123.08 (18)
C10—C14—H14A	120.5	O1—C9—C10	121.55 (17)
N3—C12—C13	122.83 (19)	N2—C9—C10	115.38 (16)
N3—C12—H12A	118.6	H2A—O2—H2B	107 (2)

### Hydrogen-bond geometry (Å, °)

**Table:**

*D*—H···*A*	*D*—H	H···*A*	*D*···*A*	*D*—H···*A*
O2—H2A···N3	0.85 (2)	2.05 (2)	2.886 (2)	168 (2)
O2—H2B···O1^i^	0.82 (3)	2.47 (3)	3.118 (2)	136 (2)
O2—H2B···N1^i^	0.82 (3)	2.43 (3)	3.197 (2)	156 (3)
N2—H2···O2^ii^	0.86	2.18	2.982 (2)	155

Symmetry codes: (i) −*x*, −*y*+1, −*z*−1; (ii) *x*, −*y*+1/2, *z*−1/2.
